# Association between iron status and incident coronary artery disease: a population based-cohort study

**DOI:** 10.1038/s41598-022-22275-0

**Published:** 2022-10-19

**Authors:** Shuren Guo, Xiaohuan Mao, Xiaohua Li, Huan Ouyang

**Affiliations:** 1 Department of Clinical Laboratory, Key Clinical Laboratory of Henan Province, The First Affiliated Hospital of Zhengzhou University, Zhengzhou, Henan People’s Republic of China; 2 Department of Clinical Laboratory, Henan Provincial People’s Hospital, People’s Hospital of Zhengzhou University, Zhengzhou, Henan People’s Republic of China; 3 Department of Clinical Laboratory, ShenQiu People’s Hospital, ShenQiu, Henan People’s Republic of China

**Keywords:** Biochemistry, Biomarkers

## Abstract

Disorders of iron metabolism has been implicated in cardiovascular disease. However, the association of serum iron stores and coronary artery disease (CAD) remains inconsistent. Here, we investigated the associations of serum iron metabolism with the incidence of CAD, the severity of coronary artery stenosis, metabolic biomarkers, and the risk of major adverse cardiovascular event (MACE). A total of 643 CAD patients and 643 healthy controls were enrolled to assess the associations of serum iron status with the presence of CAD, the severity of CAD, and the risk of MACE. Serum iron metabolism and other metabolic markers were measured in all subjects. All statistical analyses were analyzed using SPSS22.0 software and STATA statistical package. Serum level of iron metabolism markers, including serum iron, unsaturated transferrin iron binding capacity (UIBC), Total iron binding capacity (TIBC) levels, in CAD groups was significantly lower than the control group (*P* < 0.001). UIBC and TIBC were negatively correlated with ferritin in both sexes. Each unit increase of serum iron and TIBC were found to have a protective role for CAD in women (iron: OR 0.794, 95% CI (0.647–0.973), TIBC: OR 0.891, 95% CI (0.795–0.999), P < 0.05). However, high ferritin level was significant associated the CAD incident in both sexes (OR 1.029, 95% CI (1.002–1.058) in men, OR 1.013, 95% CI (1.0–1.025) in women, P < 0.05). Serum iron metabolism markers exhibited no significant association with the severity of CAD. Increased serum level of iron and TIBC levels were found to have a protective role for CAD in women, but not in men. Elevated serum ferritin is independently and positively associated with CAD in men and women.

## Introduction

Iron plays an important role in several fundamental biological processes such as erythropoiesis and cell metabolism. Iron status is a modifiable feature associated with cardiovascular disease. The role of body iron indices has been reviewed in the pathogenesis of coronary artery disease (CAD)^1–5^. Iron metabolism disorders, either deficiency or overload, were associated with increased cardiovascular morbidity and mortality^6,7^. Iron overload was positively correlated with the risk factors of cardiovascular disease, such as the risk of metabolic syndrome^8^, insulin resistance (IR)^9,10^ and new-onset type 2 diabetes mellitus^11^. Excessive iron accumulation accelerates the formation of atherosclerosis through several putative mechanisms. Firstly, iron catalyzes Fenton reaction to produce reactive oxygen species (ROS). ROS promote LDL peroxidation and induce endothelial dysfunction by reducing the bioavailability of nitric oxide^4,12–17^. ROS increased the expression of LOX-1 receptor on endothelial cells, resulting in mitochondrial DNA damage and autophagy activation^18^. Secondly, accumulated iron in adipocytes leads to adipocyte IR by increasing lipolysis and by decreasing insulin-stimulated glucose transport^7^. Adipocyte IR accelerates atherosclerosis^19^.

There were lots of studies supported that iron overload was positively correlated with CAD incident^9,20–24^. Sullivan proposed that reduced iron stores can protect against ischemic heart disease^25^. Salonen et al. observed that a higher level of iron is a risk factor for myocardial infarction in Finnish men^26^. Previous researchers have tried to reduce blood iron content in various ways to decrease the CAD incident. It has been reported that reducing iron stores through phlebotomy could decelerate the progression of atherosclerotic plaque^13,27^. Iron chelation in patients with CAD has been shown to be associated with improved endothelial function^28^.

However, some studies found no evidence that reducing iron storage can prevent cardiovascular disease, and the results of others were even contrary to the hypothesis^27,29–32^. Iron deficiency was associated with an increased risk for CAD and had detrimental effects in patients with CAD^6,32^. Weather reduced iron can protect CAD incident or not, and to what level iron should be lowered to reduce CAD risk need further study. So we conducted a case–control study enrolled a total of 643 CAD patients and 643 healthy controls to analyze the associations of serum iron levels with the presence of CAD, the severity of coronary artery stenosis, and major adverse cardiovascular event (MACE) after revasculation. MACE included ischemic stroke, myocardial infarction, and hospitalization for heart failure.

## Subjects and methods

### Study population

The control group consisted of 643 (male/female 381/262) healthy persons without cardiovascular disease via physical examination and electrocardiogram. The controls were frequency-matched to the cases on age and sex. The CAD group consisted of 643 patients, 349 men and 294 women. All CAD patients underwent physical examination and review of their medical history. Patients were excluded if they (1) had heart failure, acute myocardial infarction, coronary bypass surgery or angioplasty, coronary spasm, or myocardial bridge; (2) had cardiac diseases such as cardiomyopathy, valvular or congenital heart disease, arrhythmia; (3) patients who had malignant tumors, acute or chronic infection, iron deficiency anemia, digestive system disease, fever, connective tissue disease, autoimmune disease; (4) patients whose Blood pressure ≥ 180/110 mmHg after taking standard antihypertensive drugs, severe hepatic or renal dysfunction; (5) A history of major surgical trauma, pregnancy, mental illness, or any other cause of active blood loss within 2 weeks. The Zhengzhou University ethics Committee approved this study (The approval number 2020-KY-172). Informed consent was obtained from all participants. All the investigations were performed in accordance with the principles of the Declaration of Helsinki.

### Methods

All subjects underwent anthropometrical evaluation with measurements of weight, height, and body mass index (BMI). Smoking was defined as at least 1 cigarette per day for more than 1 year. Drinker was defined as alcohol intake of at least 90 or 45 g of liquor per day for more than 1 year for men or women, respectively.

#### Laboratory determinations

All blood samples were obtained after overnight fasting. Fasting plasma glucose (FPG), low-density lipoprotein cholesterol (LDL-C), high-density lipoprotein cholesterol (HDL-C), total cholesterol (TC), triglycerides (TG), high sensitive C-reactive protein (hs-CRP), iron metabolism biomarkers, liver function and renal function markers were measured on a Cobas 8000 Analyzer (Roche Diagnostics, Germany) using Roche Reagent following the manufacturer’s manual.

FPG was measured by the hexokinase method; TG, TC, LDL-C and HDL-C, Alanine aminotransferase (ALT) and aspartate aminotransferase (AST), creatinine, urea nitrogen (UREA), uric acid (UA) concentrations were evaluated by enzymatic methods. Hemoglobin A1c (HbA1c) was assayed by high-performance liquid chromatography on a Primer-Premier Hemoglobin Testing System (reagents were supplied by Primus). hs-CRP, homocysteine (Hcy), Apolipoprotein A (ApoA), Apolipoprotein B (ApoB) and serum ferritin levels were measured using an immune-turbid metric assay. The serum iron and unsaturated transferrin iron binding capacity (UIBC) was measured using a colorimetric test. Total iron binding capacity (TIBC) equaled the serum iron levels plus serum UIBC levels. Iron deficiency was defined as: ferritin < 100 ng/mL or 100–300 ng/mL with transferrin saturation (Tfs) < 20%^33^. Finally, estimated glomerular filtration rate (eGFR) was computed by using the chronic kidney disease epidemiology (CKD-EPI) collaboration equation^34^. Selective cardioangiography (CAG) was performed in all patients using the standard Judkins technique. The localization of coronary artery disease and the rate of lumen stenosis were determined by CAG. Structured interviews with a standardized questionnaire, including the demographics, Diabetes management, vascular risk factors (such as smoking and drinking status), and medical history, were performed by trained investigations.

### Follow-up and study outcomes

The primary outcome was the first occurrence of major adverse cardiovascular events (MACE), including ischemic stroke, myocardial infarction, and hospitalization for heart failure. Patients were followed up starting from the index date (at the health screening examination date) until two years after revasculation.

### Statistical analysis

Continuous variables were expressed as mean ± standard deviation (normally distributed data). Categorical variables are expressed as the frequency and its percentage. Continuous variables were analyzed using Student's t-test in normally distributed data, and Mann–Whitney test in non-normally distributed data. Chi-squared test was utilized for categorical variables. The association between continuous variables was assessed by Pearson correlation. The association of iron metabolism biomarkers with CAD was analyzed by logistic regression models with three progressive degrees of adjustment. Model 1 was a crude model without any confounders; model 2 was adjusted for age and cardiovascular risk factors including smoking habit, alcohol drinking habit, body mass index (BMI), hypertension, and dyslipidemia; model 3 was additionally adjusted for laboratory tests including HbA1C, ALT, AST, TC, TG, hs-CRP, eGFR. To avoid collinearity, we did not include iron and transferrin saturation into the multiple liner regression models because these two variables lied into the same causal pathway between ferritin and CAD. All the statistical analyses were executed using Statistical Package for Social Science (SPSS, version 22.0) and STATA statistical package (version 13, Texas, USA).

## Results

### Clinical characteristics

**Figure 1:**
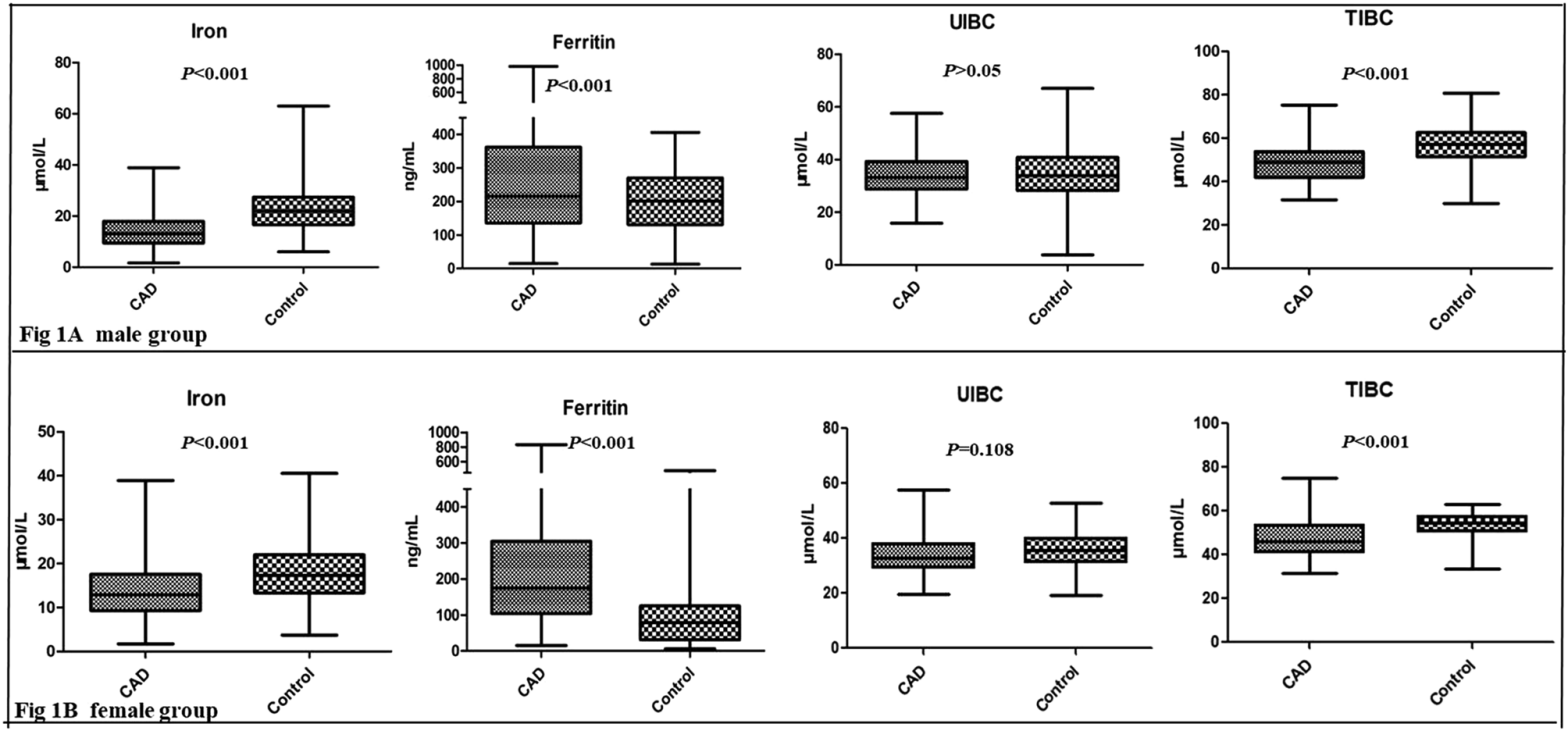
Serum iron metabolism markers.

Table 1 showed the general characteristics of the study groups according to the sexual groups. There was no significant difference in age and diastolic blood pressure (DBP) between CAD group and controls. Overall, CAD patients were more likely to be smokers, alcohol drinkers, obese, hypertensive, dyslipidemia and hyperglycemia. Comparing to the controls, individuals in the male CAD group showed significantly lower plasma concentrations of Total proteins (TP), Albumin (ALB), ApoA, Tfs and serum iron levels. While blood pressure, body mass index (BMI) and the plasma concentrations of ferritin, HbA1c, fasting blood glucose (FBG), hs-CRP, liver function and renal function markers were significantly higher in the CAD group compared to the control (*P* < 0.05) (Table 1, Fig. 1). Additionally, significant differences were found in the diabetes management styles between male and female patients with CAD (Supplement Table S1).

**Table 1:** Clinical characteristics of patients and controls.

	Male			Female		
	Control (n = 381)	CAD (n = 349)	*P*	Control (n = 262)	CAD (n = 294)	*P*
Age (years)	56.05 ± 10.75	58.56 ± 10.82	0.052	58.45 ± 9.93	60.55 ± 9.36	0.060
BMI (kg/m2)	22.99 ± 2.46	24.23 ± 2.55	0.095	23.08 ± 2.21	24.18 ± 2.68	**0.008**
SBP (mmHg)	120.32 ± 9.28	127.92 ± 17.07	** < 0.001**	120.66 ± 9.5	130.68 ± 16.93	** < 0.001**
DBP (mmHg)	74.84 ± 9.04	78.76 ± 11.14	** < 0.001**	74.24 ± 8.90	78.45 ± 11.5	** < 0.001**
HR (heart ratio)	70.21 ± 5.08	77.45 ± 10.47	** < 0.001**	70.63 ± 4.83	77.66 ± 10.22	** < 0.001**
Smoking status (%)	60 (15.75)	118 (33.81)	** < 0.001**	29 (11.07)	31 (10.54)	0.842
Drinker (%)	56 (14.7)	101 (28.94)	** < 0.001**	27 (10.31)	24 (8.16)	0.382
HbA1C (%)	5.4 ± 0.45	6.09 ± 1.21	** < 0.001**	5.52 ± 0.53	6.29 ± 1.36	** < 0.001**
FPG (mmol/L)	5.06 ± 0.6	5.33 ± 1.79	** < 0.001**	5.11 ± 0.78	5.58 ± 1.88	** < 0.001**
ALT(U/L)	24.31 ± 17.02	42.57 ± 125.79	**0.021**	18.5 ± 10.7	42.54 ± 236.76	0.303
AST(U/L)	22.22 ± 11.71	32.17 ± 38.03	** < 0.001**	21.67 ± 8.56	57.01 ± 437.43	0.416
TBIL (µmol/L)	12.99 ± 5.27	12.19 ± 8.66	0.345	10.23 ± 4.13	9.72 ± 5.49	0.395
BILD (µmol/L)	5.53 ± 1.85	5.4 ± 4.34	0.828	4.62 ± 1.63	4.75 ± 2.69	0.590
GGT(U/L)	30.31 ± 18.19	32.89 ± 27.75	0.206	23.05 ± 20.92	26.23 ± 18.86	0.172
TP(g/L)	72.86 ± 4.09	65.46 ± 5.41	** < 0.001**	74.09 ± 4.03	67.02 ± 5.58	** < 0.001**
ALB(g/L)	47.32 ± 2.82	40.94 ± 4.02	** < 0.001**	46.79 ± 2.47	41.31 ± 4.18	** < 0.001**
ALP(U/L)	68.85 ± 14.57	71.29 ± 20.42	0.056	77.92 ± 22.81	77.06 ± 25.41	0.768
TC (mmol/L)	4.64 ± 0.99	3.41 ± 0.88	** < 0.001**	4.82 ± 1	3.9 ± 1.08	** < 0.001**
TG (mmol/L)	1.74 ± 1.07	1.49 ± 1.03	0.094	1.44 ± 0.77	1.58 ± 1.25	0.403
HDL-C (mmol/L)	1.2 ± 0.29	0.99 ± 0.24	** < 0.001**	1.39 ± 0.29	1.13 ± 0.31	** < 0.001**
LDL-C (mmol/L)	2.91 ± 0.87	1.95 ± 0.79	** < 0.001**	2.95 ± 0.92	2.23 ± 0.89	** < 0.001**
APOA (g/L)	1.37 ± 0.26	1.11 ± 0.26	** < 0.001**	1.40 ± 0.24	1.17 ± 0.26	**0.003**
APOB (g/L)	0.86 ± 0.27	0.84 ± 0.36	0.727	0.86 ± 0.22	0.84 ± 0.37	0.525
Lpa (g/L)	0.16 ± 0.17	0.33 ± 1.78	0.404	0.16 ± 0.18	0.24 ± 0.26	**0.042**
homocysteine (umol/L)	13.59 ± 9.17	15.58 ± 9.08	**0.01**	12.15 ± 7.05	15.89 ± 8.56	** < 0.001**
hsCRP (mg/L)	1.61 ± 1.61	5.68 ± 14.05	**0.002**	1.57 ± 0.83	8.88 ± 26.34	**0.010**
CREA (μmol/L)	80.77 ± 12.4	87.44 ± 81.8	0.183	65.44 ± 14.7	67.46 ± 24.14	0.438
Urea (mmol/L)	5.1 ± 1.23	6 ± 3.24	** < 0.001**	4.9 ± 1.23	5.37 ± 1.98	**0.007**
UA (mmol/L)	365.08 ± 89.14	319.21 ± 100.07	** < 0.001**	278.9 ± 72.51	284.58 ± 96.06	0.601
eGFR	94.17 ± 13.18	90.39 ± 18.27	**0.008**	92.36 ± 14.45	89.97 ± 19.15	0.267
IRON (µmol/L)	23.35 ± 8.46	14.14 ± 6.4	**0.001**	18.10 ± 5.46	13.42 ± 5.77	** < 0.001**
Ferritin (ng/mL)	206.22 ± 191.69	274.03 ± 191.58	** < 0.001**	111.89 ± 81.45	233.5 ± 178.27	** < 0.001**
UIBC (µmol/L)	34.57 ± 9.99	34.38 ± 7.57	0.804	35.43 ± 6.3	34.09 ± 7.17	0.108
TIBC (µmol/L)	57.14 ± 8.13	48.52 ± 8.9	** < 0.001**	53.58 ± 5.36	47.51 ± 8.47	** < 0.001**
Tfs (transferrin saturation, %)	41.15 ± 14.23	28.7 ± 11.33	**0.009**	32.02 ± 9.99	27.89 ± 10.30	0** < 0.001**
Iron deficiency		64 (18.33%)			75 (25.5)	**0.028**

*SBP* systolic blood pressure, *DBP* diastolic blood pressure, *BMI* body mass index, *ALT* alanine aminotransferase, *AST* aspartate aminotransferase, *TC* total cholesterol, *TG* triglyceride, *HDL-c* high density lipoprotein-c, *LDL-c* low density lipoprotein-c, *ApoA* Apolipoprotein A, *ApoB* Apolipoprotein B, *FBG* fasting blood glucose, *HbA1c* glycosylated hemoglobin, *hs-CRP* high sensitive C-reactive protein.

Significant values are in bold.

### Correlation between ferritin and other metabolic biomarkers in CAD patients

We analyzed the correlation between ferritin and other metabolic biomarkers in CAD by Pearson correlation. We totally tested the correlation between serum ferritin and 16 biomarkers, i.e. blood lipids (LDL-C, HDL-C, TC, TG, ApoA, ApoB and Lp(a)), blood glucose (HbA1c), blood pressure (systolic blood pressure, SBP and diastolic blood pressure, DBP), eGFR, proinflammatory measures (hs-CRP), adiposity measure (BMI) and other iron metabolism markers. TC, TG, LDL-C and Tfs were positively correlated with ferritin in men. HDL-C, ApoA, and eGFR were negatively correlated with ferritin in women, while BMI was positively associated with ferritin in women (Table 2). UIBC and TIBC were negatively correlated with ferritin in both sexes. Interestingly, HbA1C level was negatively correlated with ferritin in men, while inversely associated with ferritin in women.

**Table 2:** Correlation between ferritin and other metabolic variables in CAD patients.

Variables	Male (n = 349)		Female (n = 294)	
	*r*	*P*	*r*	*P*
eGFR	0.023	0.706	− 0.267	** < 0.001**
**Blood lipids and glucose**				
TC (mmol/L)	0.131	**0.003**	− 0.075	0.154
TG (mmol/L)	0.198	** < 0.001**	0.085	0.105
HDL (mmol/L)	− 0.033	0.450	− 0.196	** < 0.001**
LDL (mmol/L)	0.107	**0.014**	− 0.059	0.263
APOA(g/L)	− 0.066	0.254	− 0.168	**0.008**
APOB (g/L)	0.053	0.360	0.077	0.222
Lpa (g/L)	− 0.032	0.584	0.078	0.195
HbA1C (%)	− 0.085	**0.041**	0.188	** < 0.001**
**Blood pressure**				
SBP (mmHg)	0.069	0.095	0.069	0.167
DBP (mmHg)	0.029	0.486	0.059	0.234
**Inflammation**				
hsCRP(mg/L)	0.049	0.541	− 0.089	0.341
**Adiposity**				
BMI (m/kg^2^)	0.041	0.324	0.115	**0.02**
**Iron metabolic markers**				
IRON (µmol/L)	0.081	0.051	− 0.216	** < 0.001**
UIBC (µmol/L)	− 0.262	** < 0.001**	− 0.295	** < 0.001**
TIBC (µmol/L)	− 0.168	** < 0.001**	− 0.393	** < 0.001**
Tfs (transferrin saturation, %)	0.128	**0.002**	− 0.096	0.054

Pearson correlation was performed with ferritin and other metabolic variables in CAD patients. Continuous variables with skewed distributions (ferritin, TG, hs-CRP) were log transformed for analysis.

Bold values indicate significant results with *P* < 0.05.

*eGFR* estimated glomerular filtration rate, *BMI* body mass index, *HbA1c* glycosylated haemoglobin, *TC* total cholesterol, *LDL-C* low-density lipoprotein cholesterol, *HDL-C* high-density lipoprotein cholesterol, *TG* triglycerides, *SBP* systolic blood pressure, *DBP* diastolic blood pressure, *hs-CRP* high sensitive C-reactive protein.

### Logistic regression analysis of iron metabolic markers and the risk of CAD and MACE risk

**Figure 2:**
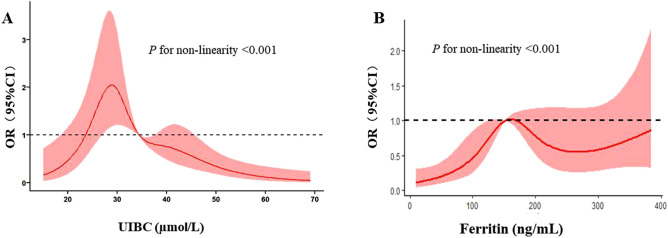
Spline model with four knots.

To illustrate the continuous relationships between parameters of iron metabolism and the risk of CAD, we assessed the concentration-risk relationship between serum ferritin levels and CAD risk by multivariate random-effects meta-regression based on the restricted cubic spline model with four knots (Fig. 2). Visual inspection revealed J-shaped relationships between ferritin and the ORs for the risk of CAD.

From an unadjusted multivariable logistic regression analysis, each unit increase in ferritin was associated with a 1.003-fold (95% confidence interval (CI), 1.002–1.004) and 1.01-fold (95% CI, 1.000–1.01) increased Odds Ratio (OR) of CAD in male and female groups respectively (Table 3). Each unit increase of serum iron level and Tfs were found to have decreased OR for CAD in both sexes (iron OR 0.83, 95% CI 0.803–0.858 in men, OR 0.894, 95% CI 0.860–0.929 in women, Tfs OR 0.896, 95% CI 0.869–0.911 in men, OR 0.943, 95% CI 0.921–0.967 in women, *P* < 0.05, Table 3). Increased serum level of TIBC had significantly lower risks for CAD in women (OR 0.943, 95% CI (0.921–0.967), *P* < 0.001, Table 3).

**Table 3:** Associations of iron metabolic markers and CAD.

Models	MALE (Control/CAD 381/349)		Female (Control/CAD 262/294)	
	OR (95% CI)	*P*	OR (95% CI)	*P*
Model 1				
Iron (µmol/L)	0.83 (0.803–0.858)	** < 0.001**	0.894 (0.860–0.929)	** < 0.001**
Ferritin (ng/mL)	1.003 (1.002–1.004)	** < 0.001**	1.01 (1–1.01)	**0.017**
UIBC (µmol/L)	1.008 (0.989–1.027)	0.417	0.97 (0.94–1.01)	0.109
TIBC (µmol/L)	0.961 (0.825–1.12)	0.613	0.9 (0.87–0.94)	** < 0.001**
Tfs (transferrin saturation, %)	0.896 (0.869–0.911)	** < 0.001**	0.943 (0.921–0.967)	** < 0.001**
**Model 2**				
Iron (µmol/L)	0.841 (0.805–0.878)	** < 0.001**	0.888 (0.847–0.931)	** < 0.001**
Ferritin (ng/mL)	1.005 (1.003–1.007)	** < 0.001**	1.010 (1.007–1.013)	** < 0.001**
Tfs (transferrin saturation, %)	0.929 (0.909–0.949)	** < 0.001**	0.936 (0.906–0.967)	** < 0.001**
UIBC (µmol/L)	–	–	–	–
TIBC (µmol/L)	–	–	0.882 (0.842–0.924)	** < 0.001**
**Model 3**				
Iron (µmol/L)	0.889 (0.626–1.264)	0.513	0.794 (0.647–0.973)	**0.026**
Ferritin (ng/mL)	1.029 (1.002–1.058)	**0.037**	1.013 (1–1.025)	**0.043**
Tfs (transferrin saturation, %)	0.87 (0.695–1.089)	0.223	0.977 (0.896–1.066)	0.599
UIBC (µmol/L)	–	–	–	–
TIBC (µmol/L)	–	–	0.891 (0.795–0.999)	**0.048**

Model 1 is crude model without any onfounders.

Model 2 was adjusted for age, BMI, SBP, DBP, Heart ratio, smoking habit, alcohol drinking habit, hypertension, diabetes status, dyslipidemia, medicine and family history of CAD.

Model 3 additionally adjusted for HbA1C, ALT, AST, TCHO, TG, hsCRP, eGFR.

Significant values are in bold.

When further adjusted for age, BMI, SBP, DBP, HR, smoking habit, alcohol drinking habit, hypertension, diabetes status, dyslipidemia, medicine and family history of CAD (model 2), the effect modification by serum iron, ferritin levels and Tfs on the CAD risk remained similar significant in both sexes. Additional adjustment for laboratory tests including HbA1C, ALT, AST, TC, TG, hs-CRP, eGFR (model 3) there was no significant direct correlation for serum iron and Tfs on the CAD risk in men (*P* > 0.05). Increased serum level of iron (OR 0.794, 95% CI (0.647–0.973), and TIBC (OR 0.891, 95% CI (0.795–0.999) were found to have a protective role for CAD in women (P < 0.05, Table 3). The OR for ferritin was significant in the both sexes (OR 1.029, 95% CI (1.002–1.058) in men, OR 1.013, 95% CI (1.00–1.025) in women, *P* < 0.05, Table 3). Regrettably, there was no statistical significance between the correlation of iron metabolism markers and MACE after calibrating for confounders (model 3, *P* > 0.05, Table 4).

**Table 4:** Associations of iron metabolic markers and the risk of MACE.

Models	Male (Control/CAD 381/349)		Female (Control/CAD 262/294)	
	OR (95% CI)	*P*	OR (95% CI)	*P*
**Model 1**				
Iron (µmol/L)	0.944 (0.917–0.973)	** < 0.001**	0.8 (0.64–1.01)	0.059
Ferritin (ng/mL)	1.001 (1–1.003)	**0.026**	1 (0.996–1.01)	0.276
UIBC (µmol/L)	0.997 (0.973–1.022)	0.839	1.04 (0.96–1.13)	0.369
TIBC (µmol/L)	0.961 (0.939–0.983)	**0.001**	0.96 (0.94–0.99)	**0.01**
Tfs (transferrin saturation, %)	0.977 (0.961–0.994)	**0.008**	0.977 (0.961–0.994)	0.155
Iron deficiency	0.882 (0.509–1.53)	0.665	0.882 (0.509–1.53)	0.67
**Model 2**				
Iron (µmol/L)	0.95 (0.92–0.981)	**0.002**	0.96 (0.91–1)	0.052
Ferritin (ng/mL)	1.001 (1–1.003)	**0.027**	1.001 (0.999–1.002)	0.526
Tfs (transferrin saturation, %)	0.98 (0.963–0.998)	**0.032**	–	–
UIBC (µmol/L)	–	–	0.99 (0.96–1.03)	0.662
TIBC (µmol/L)	0.967 (0.943–0.991)	**0.007**	0.971 (0.944–0.999)	**0.043**
Iron deficiency	–	–	0.982 (0.959–1.006)	0.141
**Model 3**				
Iron (µmol/L)	0.972 (0.916–1.031)	0.345	1.032 (0.924–1.154)	0.574
Ferritin (ng/mL)	1.001 (0.998–1.003)	0.576	–	–
Tfs (transferrin saturation, %)	0.979 (0.945–1.013)	0.226	–	–
TIBC (µmol/L)	1.009 (0.959–1.062)	0.733	1.015 (0.947–1.088)	0.674

Model 1 is crude model without any confounders.

Model 2 was adjusted for age, BMI, SBP, DBP, Heart ratio, smoking habit, alcohol drinking habit, hypertension, diabetes status, dyslipidemia, medicine and family history of CAD.

Model 3 additionally adjusted for HbA1C, ALT, AST, TC, TG, hs-CRP, eGFR.

Significant values are in bold.

### Association of iron metabolism markers with the severity of CAD

The severity of CAD was quantified by the modified Gensini scores based on the number and the extent of lesions in coronary arteries^35^. The detail calculation of modified Gensini scores was described in our previous study^36^. Gensini score of coronary artery equals the sum of all segment scores^37^. Each segment score equals segment weighting factor multiplied by a severity score. Segment weighting factor assigned to specific coronary segment are 5 for left main coronary artery, 2.5 for proximal left anterior descending coronary artery (LAD) and proximal left circumflex branch, 1.5 for mid-segment of LAD, 0.5 for second diagonal branch and posterolateral branch, and 1 for other branches. Severity score allocated to the definite percentage luminal diameter reduction are 1 for 0–25%, 2 for 26–50%, 4 for 51–75%, 8 for 76–90%, 16 for 91–99%, and 32 for 100% stenosis. Serum iron metabolism markers exhibited no significant association with the severity of CAD (Supplement Table S2).

## Discussion

Although iron status was implicated in cardiovascular disease, the relationship between iron states and CAD has long been a controversial topic in the literature. This study was conducted to assess the association between serum iron metabolism markers and CAD. For physiological reasons, the reference intervals for iron metabolism markers in women are different from men; we analyzed the association of iron status and CAD stratified by gender. In a cohort of 643 CAD patients and 643 controls, iron imbalance, as characterized by either high serum ferritin or low iron levels, was associated with an increased risk of CAD.

Overall, our findings suggested that increased serum level of iron and TIBC levels showed a protective role for CAD in women, but not in men. Iron is an essential mineral, which participates in different functions of the organism under physiological conditions. Numerous biological processes, such as oxygen transport and lipid metabolism, protein production, cellular respiration, and DNA synthesis, require the presence of iron^38^. Iron depletion may thus lead to the impaired function of different tissues including central nervous system, muscle tissue, the myocardium, the immune system and the thyroid gland. Maintaining iron metabolism is important for cardiovascular health as its high energy consumption and high mitochondrial activity^39^. Intravenous iron administration in acute myocardial infarction (MI) exerted beneficial effects in MI patients^40^.

However, we did not find iron deficiency was associated with increased risk for CAD, which was inconsistent with some previous report. The patients’ status and comorbidities might explain the inconsistence. In our study, we enrolled low percentage (less than 30%) of iron deficiency in the patient group, and we exclude the CAD patients with clinical anemia. However, there was a high prevalence of iron deficiency in acute coronary syndrome and its association with poor outcome^41^. Moreover, the definition of iron deficiency to be applied in heart disease remains controversial. The most frequently used definition of iron deficiency in patients with cardiovascular disease is ferritin < 100 μg/L or ferritin 100–299 μg/L and transferrin saturation (TSAT) < 20%^41^.

Iron is a trace element that exists in serum at low concentration of mg/dL. Iron values exhibit diurnal variation depending on dietary iron intake or patient condition^42^. Various previous studies have evaluated serum ferritin instead of serum iron level. Ferritin is an iron-binding molecule that stores iron in a biologically available form, which is essential to iron homeostasis^43^. Moreover, serum ferritin is a well-known acute-phase reactant. In our study, the serum iron level was lower in CAD group, but the ferritin level in CAD group was higher than the control group. We speculated that the increase level of ferritin was mainly due to the underlying inflammation. A batch of studies have shown a similar positive correlation between ferritin and the risk of CAD^1,20,22,23,29,30,44–46^. Xu et al. found that elevated serum ferritin was independently significantly associated with carotid atherosclerosis in women (Xu et al. 2017). We also found serum ferritin was independently significantly associated with CAD in both sexes after adjusting for inflammation marker such as hs-CRP. On the other hand, some other studies demonstrated that the higher risk of CAD is not related to the serum ferritin levels^30,47^. These studies implied that ferritin might be an inflammatory marker for atherosclerosis. Most of these studiers lack complete iron metabolism markers, it was hard to tell whether high ferritin levels reflect chronic inflammation caused by hyperglycemia or indicate iron overload, which can also lead to inflammation.

There are limited data concerning serum iron and iron saturation in CAD patients and the results are also inconsistent. The discrepancies among the studies may be partly attributable to the differences in race, dietary habits, sample size and confounding factors. Within the reference range, increased serum levels of iron and TIBC were found to have a protective role for CAD in women, but not in men. Increased serum ferritin was independently associated with CAD incident in men and women. Our study indicated that reduced serum iron level could not protect CAD incident, but increased the risk of CAD in the elder female.

The main limitation of the study is its cross-sectional design; therefore, a causal relationship could not be established. In addition, the measurements of hepcidin, a well-known regulator of body iron fluxes, were not available. However, we evaluated transferrin saturation which is an important determinant of hepcidin release^48^. Moreover, the daily iron intake needs to be estimated in the future study. Finally, there is a pragmatic need to identify circulating iron biomarkers reliably characterizing iron status within tissues.

## Supplementary Information

Supplementary Table 1.

Supplementary Table 2.

## Abbreviations

UIBC: Unsaturated iron-binding capacity
TIBC: Total iron-binding capacity
TC: Total cholesterol
TG: Triglyceride
CAG: Cardioangiography
SBP: Systolic blood pressure
DBP: Diastolic blood pressure
BMI: Body mass index
LDL-C: Low-density lipoprotein cholesterol
HDL-C: High-density lipoprotein cholesterol
hs-CRP: High sensitivity C reactive protein
AST: Aspartate aminotransferase
ALT: Alanine aminotransferase
γ-GT: γ-Glutamyl transferase
eGFR: Estimated glomerular filtration rate
BP: Blood pressure
Tfs: Transferrin saturation
IR: Insulin resistance
TC: Total cholesterol
UA: Uric acid
HbA1c: Hemoglobin A1c
Hcy: Homocysteine
ApoA: Apolipoprotein A
ApoB: Apolipoprotein B
TP: Total proteins
ALB: Albumin
MACE: Major adverse cardiovascular event
