# Supplementary material for: Association between iron status and incident coronary artery disease: a population based-cohort study
Source: Sci Rep. 2022 Oct 19;12:17490. doi: 10.1038/s41598-022-22275-0 (PMC9581887; doi:10.1038/s41598-022-22275-0)
Supplement: Supplementary file 1 — Supplementary Table 1. [file 41598_2022_22275_MOESM1_ESM.docx]

| Supplement Table 1 Clinical characteristics of patients with CAD | | | | | | | |
| --- | --- | --- | --- | --- | --- | --- | --- |
|  |  | Male  CAD (n=349) |  |  |  | female  CAD (n=294) | *P* |
| Hypertension n (%) |  | 132(37.82) |  |  |  | 108(36.73) | 0.77 |
| Clopidogrel n (%) |  | 6(4.55) |  |  |  | 5(4.63) | 0.97 |
| ACE inhibitors n (%) |  | 10(7.58) |  |  |  | 1(0.93) | **0.001** |
| ARB n (%) |  | 33(25) |  |  |  | 29(26.85) | 0.74 |
| β-blockers n (%) |  | 28(21.21) |  |  |  | 20(18.52) | 0.6 |
| Calcium channel blockers n (%) | | 36(27.27) |  |  |  | 30(27.78) | 0.97 |
| Diuretics n (%) |  | 9(6.82) |  |  |  | 10(9.26) | 0.48 |
| Diabetes n (%) |  | 113(32.38) |  |  |  | 85(28.91) | 0.34 |
| Diabetes management | |  |  |  |  |  |  |
| Lifestyle modification n (%) | | 15(13.27) |  |  |  | 10(11.76) | **<0.001** |
| Oral agents only n (%) | | 33(29.2) |  |  |  | 35(41.18) | **<0.001** |
| Insulin only n (%) |  | 64(56.64) |  |  |  | 30(35.29) | **<0.001** |
| Oral agents and insulin n (%) | | 1(0.88) |  |  |  | 10(11.76) | **<0.001** |
| Family History of CAD n (%) | | 106(30.37) |  |  |  | 66(22.45) | **0.024** |
| Dyslipidemia n (%) |  | 130(37.25) |  |  |  | 72(24.49) | **<0.001** |
| Statins n (%) |  | 75(57.69) |  |  |  | 42(58.33) | 0.93 |
| Rehospitalized ratio |  | 114(32.66) |  |  |  | 77(26.19) | 0.072 |
| ACE: angiotensin converting enzyme, ARB: angiotensin receptor blocker. | | | | | | | |
