# Supplementary material for: Association between iron status and incident coronary artery disease: a population based-cohort study
Source: Sci Rep. 2022 Oct 19;12:17490. doi: 10.1038/s41598-022-22275-0 (PMC9581887; doi:10.1038/s41598-022-22275-0)
Supplement: Supplementary file 2 — Supplementary Table 2. [file 41598_2022_22275_MOESM2_ESM.docx]

| Supplement Table 2 linear regression analysis of Gensini score | | | |  |
| --- | --- | --- | --- | --- |
| Models |  | β (95% CI) | *P* | |
| Iron (µmol/L) |  | -2.208(-7.165-2.748) | 0.381 | |
| Ferritin (ng/mL) |  | 4.351(-10.425-19.128) | 0.562 | |
| TIBC (µmol/L) |  | 0.432(-1.023-1.887) | 0.559 | |
| Tfs (transferrin saturation, %) | | 0.811(-1.589-3.211) | 0.506 | |
| Pearson correlation was performed with ferritin and other metabolic variables in CAD patients. Continuous variables with skewed distributions (ferritin, TG, hs-CRP) were log transformed for analysis. | | | |  |
